# Oxidation of Micro- and Nanograined UO_2_ Pellets by In Situ Synchrotron X-ray Diffraction

**DOI:** 10.1021/acs.inorgchem.1c02652

**Published:** 2022-01-19

**Authors:** Emanuele De Bona, Karin Popa, Olaf Walter, Marco Cologna, Christoph Hennig, Andreas C. Scheinost, Damien Prieur

**Affiliations:** † Helmholtz-Zentrum Dresden-Rossendorf, Institute of Resource Ecology, 01328 Dresden, Germany; ‡ European Commission, Joint Research Centre (JRC), Postfach 2340, 76125 Karlsruhe, Germany

## Abstract

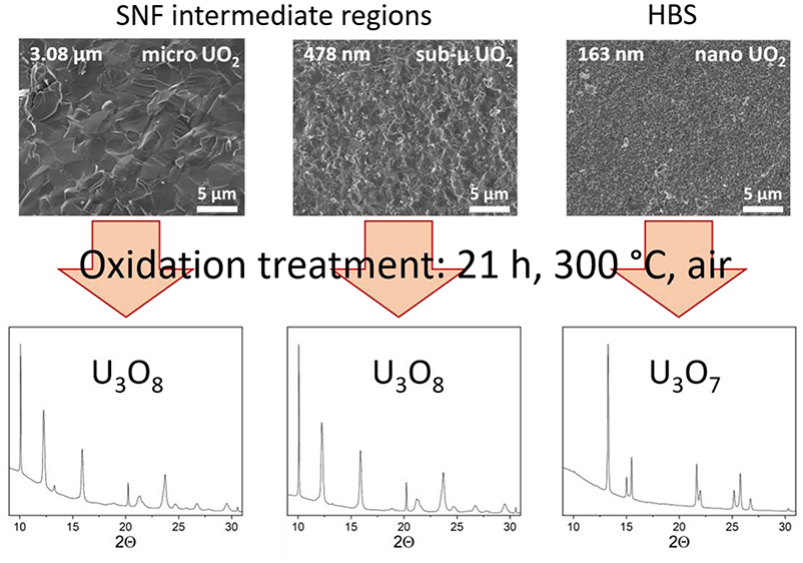

When in contact with oxidizing media, UO_2_ pellets used as nuclear fuel may transform into U_4_O_9_, U_3_O_7_, and U_3_O_8_. The latter starts forming by stress-induced phase transformation only upon cracking of the pristine U_3_O_7_ and is associated with a 36% volumetric expansion with respect to the initial UO_2_. This may pose a safety issue for spent nuclear fuel (SNF) management as it could imply a confinement failure and hence dispersion of radionuclides within the environment. In this work, UO_2_ with different grain sizes (representative of the grain size in different radial positions in the SNF) was oxidized in air at 300 °C, and the oxidation mechanisms were investigated using in situ synchrotron X-ray diffraction. The formation of U_3_O_8_ was detected only in UO_2_ pellets with larger grains (3.08 ± 0.06 μm and 478 ± 17 nm), while U_3_O_8_ did not develop in sintered UO_2_ with a grain size of 163 ± 9 nm. This result shows that, in dense materials, a sufficiently fine microstructure inhibits both the cracking of U_3_O_7_ and the subsequent formation of U_3_O_8_. Hence, the nanostructure prevents the material from undergoing significant volumetric expansion. Considering that the peripheral region of SNF is constituted by the high burnup structure, characterized by 100–300 nm-sized grains and micrometric porosity, these findings are relevant for a better understanding of the spent nuclear fuel behavior and hence for the safety of the nuclear waste storage.

## Abstract 2

The present work reports on the oxidation behavior of UO_2_ disks having equal density but different grain size (3.08 μm, 478 nm, and 163 nm). After 21 h at 300 °C under air, the first two samples transformed into U_3_O_8_, while only U_3_O_7_ could be detected in the third one. This finding is relevant for nuclear waste management, since the grain size of the high burnup structure is comparable to that of the third sample.

## 1 Introduction

Currently, the solution favored by most countries worldwide for the direct disposal of their spent nuclear fuel (SNF) is the deep geological repository.^1^ Before this final solution, SNF is temporarily stored under wet or dry storage conditions, while decay heat gradually decreases to acceptable levels. In order to assess its safety and validate this option, a deep understanding of the long-term behavior of the whole system (SNF and its container) is required. In the case of a leakage in the container during dry interim storage, the SNF would come in contact with the external environment (i.e., air). This would lead to the formation of an oxidized layer on the SNF surface, potentially detrimental for the system integrity, enhancing the material degradation and eventually resulting in the release of radionuclides. Under oxidizing conditions, UO_2_ transforms into the more thermodynamically stable U_3_O_8_, involving a 36% volume expansion that leads to stress generation on the cladding as well as fuel degradation.^2^ Moreover, the solubility of U(V) and U(VI) in aqueous media is higher than that of U(IV), resulting in a faster dispersion of the nuclear waste residuals in the environment at a later stage if groundwater manages to come in contact with the oxidized SNF.^3^ Understanding the SNF oxidative degradation mechanism is therefore fundamental for the evaluation of the safety of its repository conditions.^4^

The oxidation of UO_2_ in different forms (powder, sintered, single crystal) and under different conditions (temperature, oxygen partial pressure, radiation field) has been studied for over 50 years, but some aspects still remain unclear.^5^ At temperatures representative of dry storage conditions (about 300 °C), the oxidation of UO_2_ to U_3_O_8_ proceeds following the reaction UO_2_ → U_4_O_9_ → β-U_3_O_7_ → U_3_O_8_.^6−9^ At high temperatures, UO_2_ has a broad hyperstoichiometric domain (UO_2+*x*_, with *x* increasing with temperature up to 0.24), where oxygen is included in the lattice and the cell gradually shrinks.^10,11^ As the oxidation continues beyond the solubility limit of oxygen in UO_2_, U_4_O_9_ starts forming. The formula U_4_O_9_ (or U_4_O_9–*y*_) is actually a simplification of the real structural formula U_256_O_572_ (stoichiometry 2.234 instead of 2.25) that describes a superstructure in which excess oxygen defects are organized in cuboctahedral clusters, whose periodicity defines the superstructure itself.^12,13^ The fluorite-related U_4_O_9_ superstructure is characterized by a cubic cell (*I*4̅3*d*) with four-fold lattice dimensions with respect to the original UO_2(+*x*)_ (*a*_U_4_O_9__ = 4*a*_UO_2+*x*__). Further oxidation introduces more oxygen cuboctahedra and induces an anisotropic distortion in the structure, leading to the formation of tetragonal U_3_O_7_ (*a*/*c* > 1),^12−15^ in a similar way to what happens in other fluorite-related anion-excess superstructures such as Ca_2_YbO_7_.^16^

At temperatures around 200–300 °C, the first stage, with the formation of U_4_O_9_ and U_3_O_7_, is characterized by a pseudoparabolic weight gain curve, typical of a diffusion-controlled process.^17^ The transformation of U_3_O_7_ into orthorhombic U_3_O_8_ is instead accompanied by a sigmoidal weight gain curve, related to a combination of a nucleation and growth mechanism^18^ and macrocracking,^19^ whose relative contribution is determined by factors such as temperature and sample characteristics (for example, the crystallite domain size). In particular, cracks start appearing after a certain incubation time, necessary to form a layer of U_3_O_7_ thick enough to generate sufficient stress due to the lattice parameter mismatch at the interface with the pristine UO_2_.^19−21^

However, SNF is an extremely complex system that differs substantially from pure unirradiated UO_2_, with notably the presence of minor actinides and fission products, nonuniformly distributed in the UO_2_ matrix, as well as microstructural gradients.^22^ During reactor operation, the high burnup structure (HBS) starts to form at the rim of the fuel pellets, characterized by the restructuring of the initial 10–15 μm grains into 100–300 nm ones (in the order of 10^5^ new grains for each original one), surrounding micrometric pores.^23−25^

Studies performed on powders showed that particles under a certain size (200 nm) did not develop U_3_O_8_ while being oxidized under air.^26,27^ Similar oxidation studies were performed also on sintered UO_2_ pellets,^19^ but never focused on the impact of the grain size on the oxidation behavior. As the grain size characteristic of the HBS also falls below the critical thickness of the U_3_O_7_ for cracking (0.4 μm),^19^ this work aims to investigate potential differences in the oxidation behavior of HBS with respect to the bulk of the fuel. Due to its chemical and microstructural inhomogeneity and its high radioactivity, the study of the oxidation of real HBS is extremely complex. A solution consists of using dedicated materials that have been designed in a way to decouple the effects of grain size, porosity, chemical composition, and self-irradiation on the oxidation of the HBS.

The microstructural aspect, and especially the grain size effect, has not been extensively explored yet, but the development of field-assisted sintering techniques (FASTs) such as spark plasma sintering (SPS) introduced new possibilities for nuclear ceramics processing.^28−31^ By controlling the processing parameters, densification can be achieved while strongly limiting coarsening, leading to the production of dense UO_2_,^32−35^ UO_2+*x*_,^36,37^ and ThO_2_^38^ of grain size comparable to that of the HBS.

In this work, high-temperature synchrotron radiation X-ray diffraction (HT-SRXRD) and X-ray adsorption near edge structure (XANES) were used to follow the isothermal oxidation of sintered UO_2_ samples of different grain sizes. The samples were prepared by applying three different SPS treatments to UO_2+*x*_ nanopowders produced by hydrothermal decomposition of U(IV) oxalate, obtaining three different final microstructures, one of which with grain size in the range of HBS.^35^ All samples were characterized by means of conventional X-ray diffraction (XRD) and scanning electron microscopy (SEM) at the Joint Research Centre (Karlsruhe, Germany) before the oxidation test, which was performed at the European Synchrotron Radiation Facility in Grenoble (France). Remarkably, U_3_O_8_ was detected in the samples having larger grain sizes (3.08 ± 0.06 μm and 478 ± 17 nm), but not in the one characterized by 163 ± 9 nm grains.

## 2 Experimental Section

### 2.1 Sample Preparation

The optimization of the sample preparation in order to obtain dense (95% TD) UO_2_ disks with final grain size close to the one of the HBS was already described in a dedicated publication.^35^ Briefly, UO_2_ nanopowders were produced by hydrothermal decomposition of U(IV) oxalate at 170 °C. To protect the operators from ingestion and/or inhalation of the powders, their synthesis (liquid route) was performed under a fumehood, and all the following processing was carried out inside gloveboxes (N_2_ or Ar, <1% vol O_2_). The SPS device used in this work is a FCT Systeme GmbH, modified for inclusion in a 1 × 1 × 1.5 m^3^ glovebox, whose nuclearization is described by Tyrpekl et al.^39^

The powders were sintered with three different treatments, whose main parameters are summarized in Table 1. Every treatment was performed in vacuum. The two-step (2S-SPS) and high-pressure SPS (HP-SPS) treatments were optimized to minimize the final grain size, by favoring densification over coarsening during sintering. SPS and 2S-SPS were performed in graphite dies, while in HP-SPS the powder was loaded into a SiC die. A final annealing under Ar–H_2_ (4% vol) at 600 °C was performed to reduce all the samples to the same UO_2.00_ stoichiometry, without affecting their microstructures (the temperature of the annealing was lower than the maximum sintering temperatures in every SPS treatment).

**Table 1:** SPS Treatments Applied on the UO_2_ Nanopowders^a^

sample	treatment	σ (MPa)	*T* (°C)	*t* (min)	*Ṫ* (°C/min)	grain size
micro UO_2_	SPS	70	1600	10	200	3.08 ± 0.06 μm
sub-μ UO_2_	2S-SPS	70	650–550	0.05–100	200	478 ± 17 nm
nano UO_2_	HP-SPS	500	660	0.5	100	163 ± 9 nm

a The sintering parameters reported in the table are the following: σ is the pressure applied onto the powder (sample diameter = 6 mm), *T* is the maximum sintering temperature, *t* is the hold time at the maximum sintering temperature, and *Ṫ* is the heating and cooling rates.

**Figure 1:**
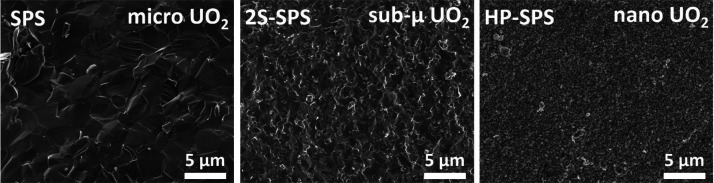
SEM SE images of the microstructures of the as-sintered materials. The grain sizes calculated with the intercept method are 3.08 ± 0.06 μm for the micro UO_2_ obtained by regular SPS (70 MPa, 10 min, 1600 °C), 478 ± 17 nm for the sub-μ UO_2_ produced by 2S-SPS (70 MPa, 3 s at 650 °C, 100 min at 550 °C), and 163 ± 9 nm for the nano UO_2_ prepared by HP-SPS (500 MPa, 30 s, 660 °C).

As a result of the different sintering conditions applied, the final microstructures exhibited different degrees of coarsening despite the samples having the same fractional porosity (5%). Figure 1 shows the SEM pictures of the fracture surfaces of the annealed UO_2_ disks. The grain size was evaluated with the standard ASTM E112-12^40^ intercept method using straight lines crossing at least 50 grain boundaries. The values obtained were 3.08 ± 0.06 μm (micro UO_2_), 478 ± 17 nm (sub-μ UO_2_), and 163 ± 9 nm (nano UO_2_). Rietveld refinement of the XRD patterns revealed a lattice parameter of 5.470 ± 0.001 Å for all samples, meaning they were successfully reduced to UO_2.00_.

### 2.2 Characterization

The as sintered samples were characterized by XRD and SEM at the European Commission Joint Research Centre in Karlsruhe.

XRD measurements of the as-produced disks were performed using a Rigaku Miniflex 600 in Bragg–Brentano geometry, with a ceramic copper source (40 kV, 15 mA) without monochromator (Kα_1_ = 1.5406 Å, Kα_2_ = 1.5444 Å), supplied with a Hy-Pix 400MF 2D HPAD detector. Samples were prepared by mechanical grinding in a paraffin suspension and then poured onto low-background Si holders. Analyses of the diffraction patterns were performed with the software Jana2006^41^ using pseudo-Voigt functions for fitting the peaks shape. The deviation from stoichiometry (*x* in UO_2+*x*_) of the samples was evaluated from the lattice parameter (*a*), as determined by Rietveld refinement of the diffraction patterns, using the formula *a* = 5.4705 – 0.132*x* proposed by Teske et al.^42^ The crystallographic data and the atomic coordinates used to fit the experimental patterns were taken from the ICSD–FIZ Karlsruhe database.^43^

SEM images were acquired with a dual-beam focused ion beam ThermoFisher Scientific (ex FEI) Versa 3D SEM with field emission gun operated at 30 keV.

### 2.3 HT-SRXRD and XANES

Combined HT-SRXRD and XANES measurements were performed at European Synchrotron Radiation Facility (ESRF, Grenoble) on the HZDR-operated Rossendorf Beamline (BM20) using the setup of the XRD-2 diffractometer.^44^ This beamline is dedicated to X-ray absorption and emission spectroscopies as well as X-ray powder diffraction (P-XRD) on actinide materials.

Samples were prepared for P-XRD by manually milling for 1 min a fragment of the sintered pellets in an agate mortar. This process yields particles with the same density as the original pellets (95% TD) and therefore more representative of a dense material than loose powders (that instead include a higher amount of porosity). Of course, having introduced the pulverization in the sample preparation, and not leaving it to the oxidation process itself, will make it impossible to compare the oxidation kinetics of this study with those of other studies on whole pellets. On the other hand, even for the samples with the largest grain size, each milled particle would still be a dense (95% TD) pellet fragment constituted of several grains, allowing us to investigate the role of the grain size on the oxide phases developed during oxidation.

**Figure 2:**
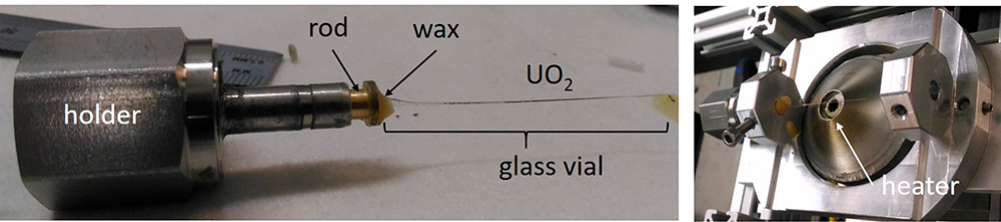
Photos of the experimental setup, with the quartz capillary containing the ground UO_2_ disks already fixed with wax to the metallic rod and mounted on the holder (left), which was then installed onto the support and fixed above the hot gas generator (right). All the components are labeled in the pictures.

About 0.3 mg of ground UO_2_ was then poured in quartz capillaries (inner diameter of 0.2 mm) open on both sides to allow gas flow. The capillaries were mounted onto the sample holder through an open metallic rod, where they were fixed with wax. This sample holder was then installed onto the support and tightened with a screw, as shown in Figure 2.

A hot gas generator from Cyberstar controlled by a Eurotherm and a gas flow controller was used to heat up the samples with a precision of ±1 °C. Figure 2 (right) shows how the support was mounted onto the heater (labeled in the Figure). Both ends of the capillaries were left open to allow for air flow. The 200 × 200 μm synchrotron beam was aligned with a macrocamera in the center of the flat temperature profile of the capillary. No direct measurement of the temperature was performed on the sample, but the system was previously calibrated with identical geometrical conditions using a standard material.

The heating rate was set at 300 °C/min in order to minimize its effect on the isothermal oxidation experiment. Each treatment was performed at 300 °C for about 21 h. The HT-XRD acquisition time was 10 s in the initial part of the experiment and gradually increased to 20 and then 60 s as the changes of the phase composition of the samples became less abrupt. Synchrotron powder X-ray diffraction data were recorded using a Pilatus3X 2Mdetector (Dectris Ltd.), with a sensitive area of 253.7 × 288.8 mm^2^ (width × height). The excitation energy was set to 17038 eV to avoid scattering background due to fluorescence above the U L3 absorption edge at 17166 eV.

U L3 XANES spectra were collected with a single-element Si drift detector (Vortex X90 CUBE, 1000 mm SDD, 50 mm^2^ collimated down to 30 mm^2^, 25 mm Be window) with a FalconX1 processor. Each XANES acquisition was 5 min long. The XRD and XANES measurements were performed successively on the same sample position to guarantee that the crystalline phases and the oxidation states are probed at the same volume increment. Both acquisitions were performed on the same sets of samples. XRD measurements were automatized using a macro, while XANES analyses required some changes in the setup and therefore the interruption of the XRD acquisitions.

## 3 Results

### 3.1 HT-SRXRD

The as-sintered materials were then oxidized in air for 21 h, and their structural evolution was monitored by HT-SRXRD. The sampling frequency during the 21 h long oxidations allowed to have a complete and comprehensive overview of the transformation ongoing in the samples. In HT-XRD, the buildup of the different compounds could be followed by the emergence or disappearance of peaks and shoulders over time or by their changes in shape and relative intensity. However, the coexistence of many phases and domains, each one developing interdependently, made it sometimes complicated to extract all the possible information.

**Figure 3:**
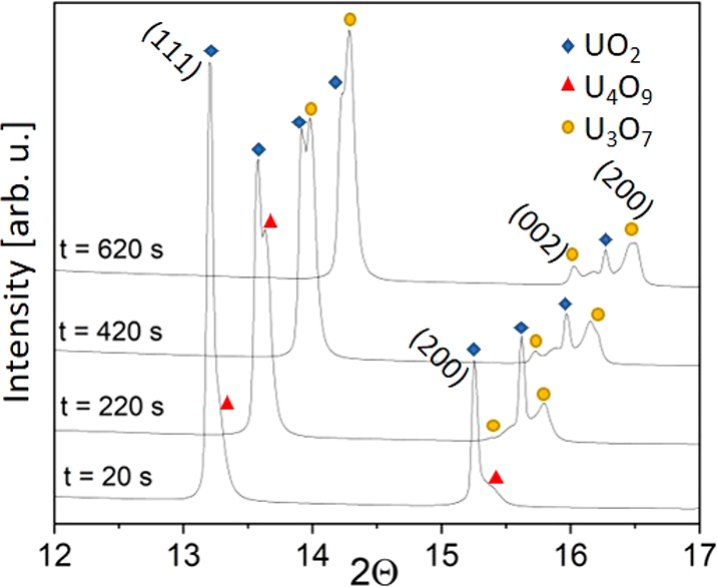
Evolution of the HT-XRD pattern of the micrograined UO_2_ sample in the first 620 s of the isothermal oxidative treatment. Patterns have been gradually shifted to higher 2Θ for the sake of readability.

An example is shown in Figure 3, with the appearance of U_4_O_9_ and U_3_O_7_ on UO_2_ in the microsized sample during the first 620 s of the oxidative treatment. Initially, UO_2_ converted into U_4_O_9_, as can be observed with the appearance of a shoulder on the right-side of the UO_2_ peaks. After 220 s, U_4_O_9_ oxidized into tetragonal U_3_O_7_, which implied the splitting of the 200 peak into the 200–002 doublet. This behavior, consistent with previous results,^6,9^ was observed in all the samples, although with different kinetics.

**Figure 4:**
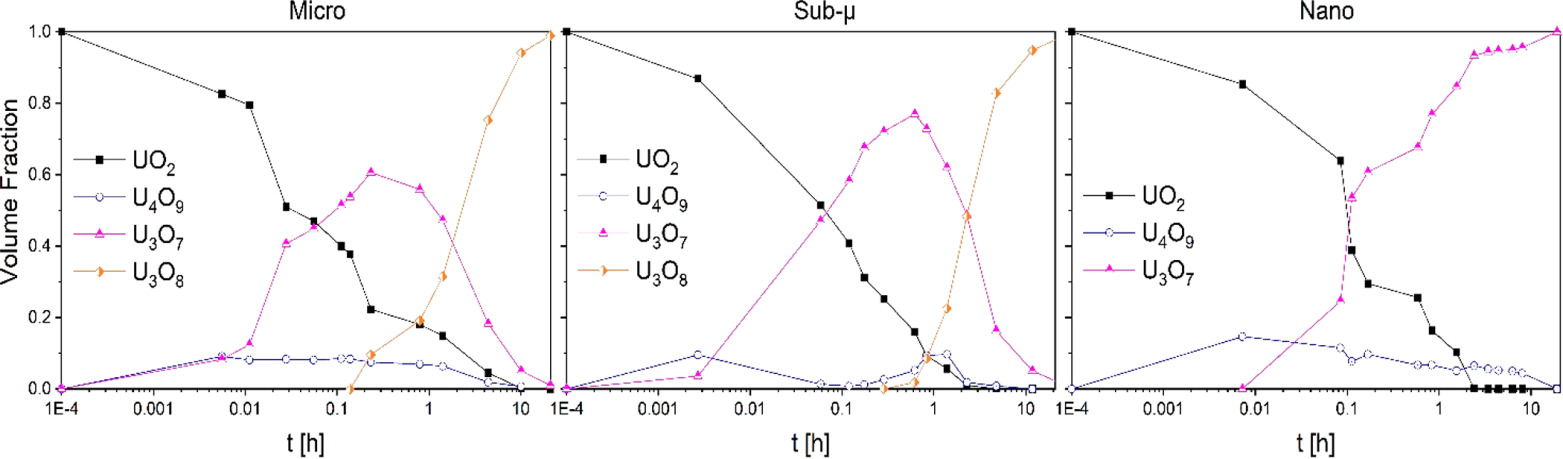
Evolution of the different phases in the three samples during the oxidative treatments (grouped by sample).

**Figure 5:**
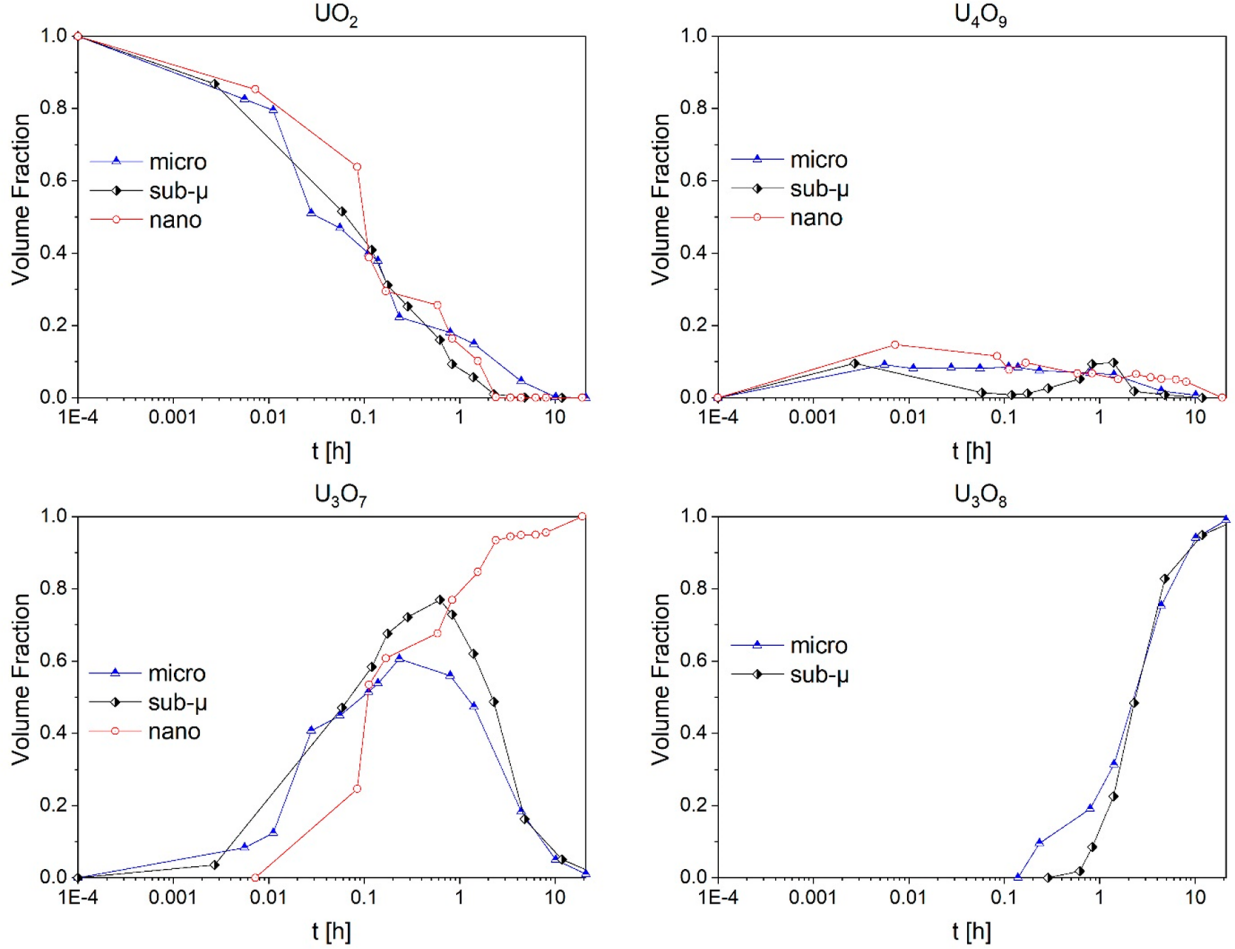
Evolution of the different phases in the three samples during the oxidative treatments (grouped by phase).

Figures 4 and 5 show the evolution of the different phases during the oxidation of the samples, grouped by sample or by phase, respectively. These volume fractions were calculated by Rietveld refinements using the crystallographic data found by Desgranges et al.^13^ As can be seen in Figure 5, in all three samples, the volumetric fraction of U_4_O_9_ remained constantly below 20%. This can be understood by its prompt conversion to U_3_O_7_. After 10 min, this latter oxide constituted the largest volume fraction for all the samples. At this point, once U_3_O_7_ was the major phase, different oxidation behaviors were observed depending on the grain size.

In the micro sample, U_3_O_8_ was detected after 15 min, which corresponded to the maximum U_3_O_7_ volume fraction (ca. 60%). Due to the large grain size, the complete conversion of the UO_2_ in the bulk of the grains to U_3_O_7_ was significantly delayed with respect to the other samples (completed after about 10 h, instead of about 2.5 h as in the sub-μ and nano samples).

In the sub-μ UO_2_ sample, the U_3_O_7_ volume fraction kept increasing up to 80% (after about 30 min), and only then it started to be consumed by its transformation into U_3_O_8_. This oxidation then proceeded at the expenses of U_3_O_7_, while in turn, UO_2_ was consumed and transformed to U_4_O_9_ and then U_3_O_7_. After about 11 h, no more U_4_O_9_ could be detected, and the sample was composed of 95% of U_3_O_8_. For both the micro and the sub-μ samples, at the end of the experiment (21 h), U_3_O_8_ was the main phase, but a small share (below 3%) of U_3_O_7_ was left. No U_3_O_8_ was detected in the nanograined sample, where all the UO_2_ was converted to U_4_O_9_ and U_3_O_7_ after 2.5 h, and U_4_O_9_ slowly disappeared throughout the rest of the oxidative treatment.

### 3.2 XANES

**Figure 6:**
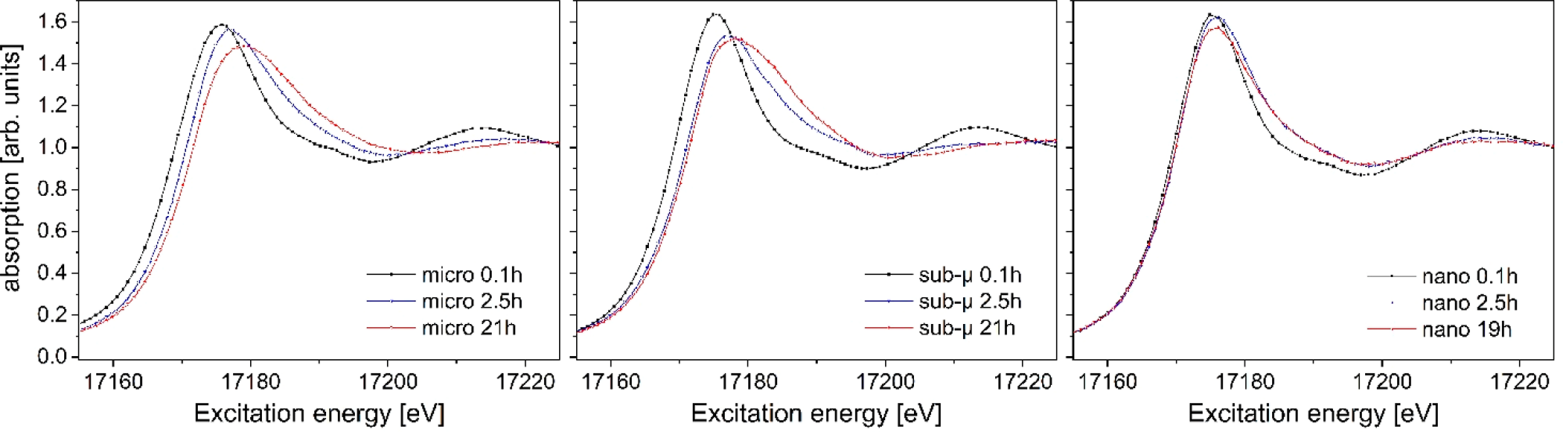
Evolution of the XANES spectra of the three samples during the oxidative treatment.

**Figure 7:**
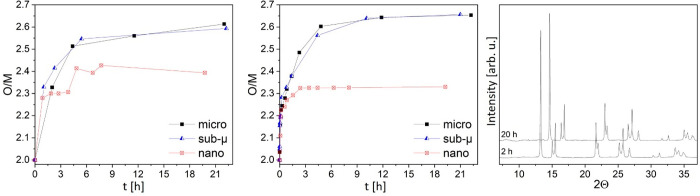
Comparison of the O/M ratios of the three samples as measured by XANES (left) and XRD (center) during the oxidative treatments. On the right, the comparison between the XRD acquired on the nano sample after 2 and 20 h (the last acquisition).

Similar to the HT-SRXRD characterization, the oxidation of the samples could be followed by the change of the XANES spectra over time. The evolution of the spectra for each sample is shown in Figure 6. Fitting these data with a combination of different U oxides references (UO_2_, U_4_O_9_, U_3_O_7_, and U_3_O_8_) allowed calculating the O/M ratios that are reported in Figure 7 (left), while the O/M ratios derived from the HT-SRXRD analyses are reported for comparison in the central block of the figure.

Although U_3_O_8_ was not observed by XRD, analyses of the XANES data highlighted that further oxidation was not completely suppressed in the nano sample either. Consistently with the results obtained by HT-SRXRD, the micro and sub-μ samples reached an O/M of about 2.6, very close to the theoretical value of 2.66 of U_3_O_8_. The nano sample instead reached O/M = 2.4 according to XANES, which is higher than the theoretical value for U_3_O_7_ (2.33), from which it departed after about 4 h. At this threshold, no new crystalline phase could be detected in the HT-SRXRD: As can be seen in the plot on the right of Figure 7, the HT-SRXRD pattern of the nano sample remained almost identical from 2 h (when U_3_O_7_ reached 90%) until the end of the treatment (20 h).

## 4 Discussion

### 4.1 Nanograins Inhibit U_3_O_8_ Formation

**Figure 8:**
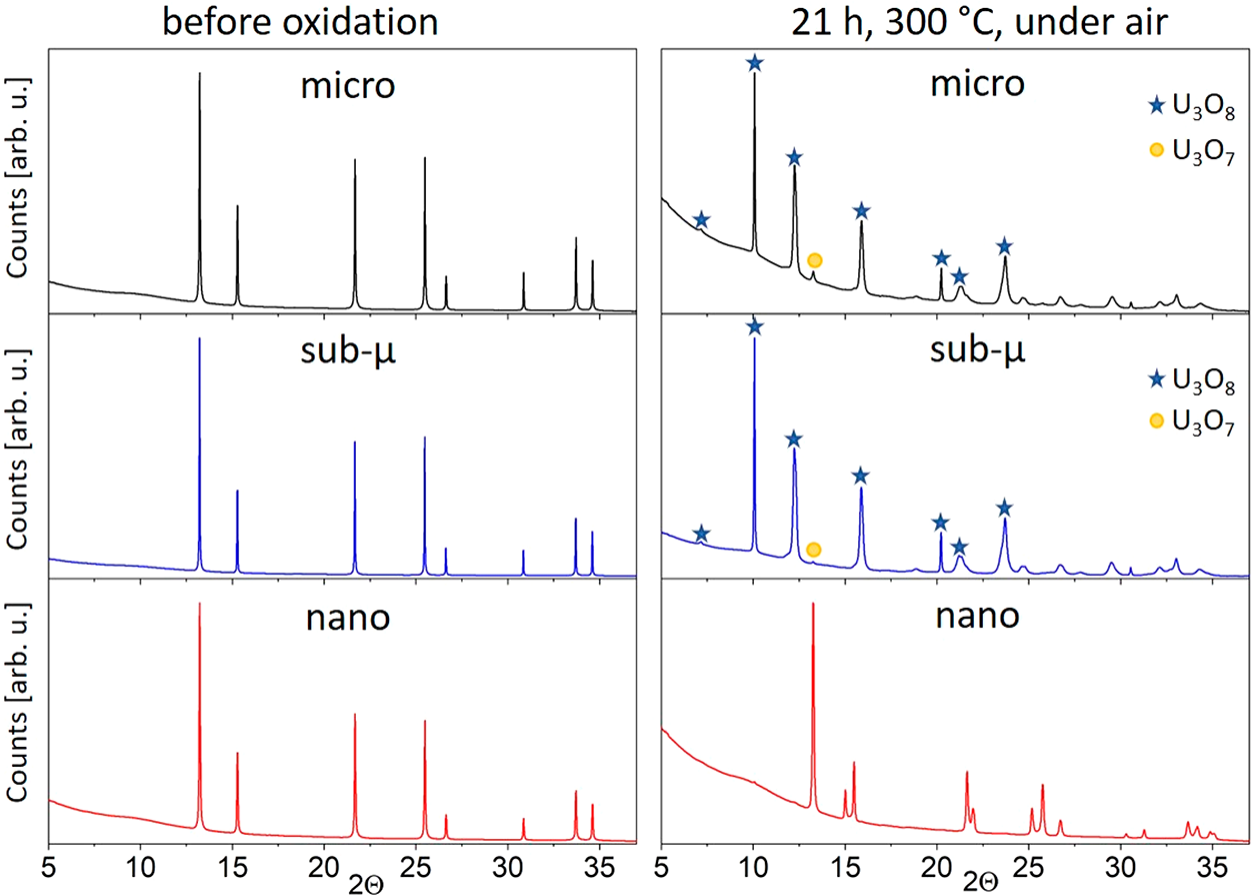
Comparison of the SR-XRD patterns for the three samples before and after the oxidation treatment. Contrary to the micro and sub-μ UO_2_ samples, no U_3_O_8_ was detected in the nanosized compounds after 21 h in air at 300 °C. The main diffraction reflexes of U_3_O_8_ are marked with a blue star.

A comparison of the diffraction patterns of the initial and final states of the samples is shown in Figure 8, where the main U_3_O_8_ peaks are marked with a blue star. Clearly, the peaks of U_3_O_8_ could not be detected in the nanograined sample. Despite the significant differences between the systems, the absence of U_3_O_8_ during oxidation of sintered nanograined UO_2_ is consistent with previous results on loose powders (with a particle size inferior to 200 nm).^19,26^ In these studies, the absence of U_3_O_8_ was accompanied by the lack of cracks on the nanopowders. In their SEM characterization, Quémard et al. did not observe cracks on nanometric powders that also did not develop U_3_O_8_ during isothermal oxidation treatments, contrary to micrometric powders, polycrystalline pellets (with grains in the micrometre range), and single crystal samples.^19^ Similar results were obtained by Leinders et al, who performed transmission electron microscopy (TEM) inspections on fine powders (<200 nm) that were oxidized up to U_3_O_7–*z*_, without finding evidence of cracking.^26^

According to the mechanism proposed by Bae et al.^45^ and Tempest et al.,^46^ both inter- and intragranular cracking play a key role in the oxidation of sintered unirradiated UO_2_ pellets to U_3_O_8_. In their observations, the formation of U_3_O_7_ on the pellets surface led first to intergranular cracks and later on to their propagation as intragranular cracks toward the grain center. U_3_O_8_ was then detected at the microcracks edges only after the onset of intragranular cracking. From this point on, the formation of U_3_O_8_, and the subsequent volume increase, resulted in the spallation of the sample and accelerated the oxidation process. In the present work, the first part of the intergranular cracking can be assumed to have already taken place, as samples were preground prior to the oxidative treatments. As the volume fraction of U_3_O_7_ increased, more intergranular cracking reasonably occurred, although this was not proven, as SEM or TEM post-mortem examination was not performed in this study.

It is now worth remembering that the transformation of UO_2_ into U_3_O_7_ (through U_4_O_9_) involves a lattice distortion, as more and more oxygen atoms are included in the lattice and reorganize into cuboctahedra. However, U_3_O_7_ needs a stabilizing factor to avoid its transformation back into a mixture of U_4_O_9_ and U_3_O_8_.^13^ Indeed, the stress generated by the oxidated layer growing on the pristine UO_2_ can act as the stabilizing factor, leading to U_3_O_7_ being actually observed both in powder and sintered samples. As the U_3_O_7_ layer topotactically grows onto the pristine UO_2_, stress localization minimizes the system energy and at the same time stabilizes the U_3_O_7_ phase.^21^ Once the U_3_O_7_ layer reaches the critical thickness around 0.4 μm, local stress becomes too high and results in cracking. At this point, the stabilizing element ceases, and U_3_O_8_ forms in a similar way to a martensitic-type transformation.^47^

In sintered samples, by definition, the crystallite size cannot exceed the grain size. Therefore, in the nanograined sample, the U_3_O_7_ domains are bound to remain well below the critical thickness for cracking, thus not leading to intragranular cracking and to U_3_O_7_ into U_3_O_8_ transformation. In the other samples instead, when the U_3_O_7_ layer grew thick enough, cracks formed and opened up the grains, while U_3_O_7_ transformed into U_3_O_8_ at the crack initiation. The volume expansion associated with the U_3_O_8_ formation contributed to crack propagation and accelerated the process of further pulverization and grain opening. It is worth noting that this mechanism prevents the formation of U_3_O_8_ and the correlated volume expansion in nanograined UO_2_, but does not necessarily imply the suppression of intergranular cracking that is responsible for the pulverization of the material.

### 4.2 Nanograins Oxidation beyond U_3_O_7_

Similar to what was reported in the literature for fine powders, oxidation proceeded to a certain extent beyond U_3_O_7_.^19,26^ It was indeed found that, especially for extremely long thermogravimetric analyses (TGA) experiments, the sample oxidation did not stop with the formation of U_3_O_7_, but rather advanced orders of magnitude slower than for micrometric powders or sintered pellets (and possibly with a different weight gain curve shape).^19^ Following TEM observations, a mechanism proposed to explain the discrepancies between the O/M ratios measured by XRD refinements and TGA during the oxidation of fine powders was the nucleation of amorphous UO_3_ nanodomains on the powder surface.^26^ A similar mechanism could have taken place in the nano sample analyzed in this work, with the UO_3_ phase remaining undetected in XRD, being amorphous, and instead being revealed by the XANES. The time frame of this experiment does not allow to draw conclusions on whether the O/M ratio of the sample reaches a plateau or if it would increase with extremely slow kinetics (such as for fine powders). It is worth noting that the kinetics of nucleation and growth of amorphous UO_3_ could differ between the boundary of a nanograin embedded in a dense material and the free surface of a loose powder nanoparticle.

## 5 Conclusions

The isothermal oxidation at 300 °C under air of UO_2_ samples with grain sizes of about 3 μm (“micro”), 460 nm (“sub-μ”), and 160 nm (“nano”) was followed by in situ HT-XRD and XANES. These samples were prepared by manual grinding of densified pellets, being therefore more representative of sintered material than loose powders. However, it must be kept in mind that the milling process increased the specific surface area of the samples (from pellet to ground particles). This affected the oxidation kinetics (as the samples were manually pulverized and did not pulverize as a consequence of the oxidation process itself), but still allowed to study the influence of the grain size on the oxide phases developed in 95% TD UO_2_ samples during oxidation.

While the micro and sub-μ samples followed a similar behavior, being almost fully converted to U_3_O_8_ after about 11 h of oxidative treatment, no U_3_O_8_ was detected in the nano sample. This could have positive implications in the safety of SNF storage, as the formation of U_3_O_8_ involves a 36% volume expansion, detrimental for the rod integrity, and is triggered by the cracking of the material. Such a finding is particularly interesting considering that the peripheral region of SNF is constituted of the HBS, whose grain size is on the order of magnitude of the nano sample used in this work.

This behavior was already reported in the literature for the oxidation of fine powders, but this work shows that it is based on the grain size, rather than on the particle size, and therefore, it is characteristic also of dense nanostructured systems. However, oxidation beyond U_3_O_7_ is not completely inhibited: XANES characterization revealed a final O/M of 2.42, significantly higher than the theoretical value of 2.33 of U_3_O_7_. In nanometric powders, the oxidation beyond U_3_O_7_ was reported to proceed with the nucleation and growth of amorphous UO_3_ nanodomains. Further investigation, especially by means of electron microscopy (both SEM and TEM) is needed to assess if this mechanism is also taking place in the case of dense materials.

This study contributes to a better understanding of the interaction of SNF with the external environment in the case of repository confinement failure. With a separate effect study approach, the results herein reported shall be combined with what was found in oxidation experiments on SIMFUELs reproducing the chemical composition of SNF. By having a better representation of the real oxidation behavior of the HBS, other variables need to be added to the system, namely the fission products and the porosity, and in a further stage the self-irradiation effect given by the long-lived minor actinides.

The in situ oxidation study of nano- to micrograin UO_2_ presented here demonstrates the fundamental role of the microstructure in the oxidation kinetics and the development of the different uranium oxide phases.
